# Obstetric outcome of female genital mutilation in the Gambia – an observational study

**DOI:** 10.4314/ahs.v22i4.44

**Published:** 2022-12

**Authors:** Patrick Idoko, Alice Armitage, Momodou T Nyassi, Lucas Jatta, Neneh Bah, Awa Jah, Dado Jabbie, Mustapha Bittaye

**Affiliations:** 1 School of Medical and Allied Health Sciences, University of The Gambia, Banjul, The Gambia; 2 Edward Francis Small Teaching Hospital, Banjul, the Gambia; 3 Medical Research Council Laboratory the Gambia; 4 Bansang Hospital, The Gambia; 5 Brikama District Hospital, The Gambia; 6 Bundung Maternal and Child Hospital, The Gambia

**Keywords:** Female genital mutilation, obstetric outcome, Gambia

## Abstract

**Background:** A 2010 survey in The Gambia among women of reproductive age put the prevalence rate of FGM/C at 76.3%. FGM/C was banned in 2015, but there is no real effort at enforcement of the ban. This study aimed to provide national data on obstetric outcomes to support advocacy and health education.

A multicentre observational study to assess the obstetric and neonatal outcomes of parturient women with and without FGM/C was carried out across 4 healthcare facilities in The Gambia. The primary outcome was postpartum haemorrhage (>500ml) and secondary outcomes were caesarean section, perineal tears (including episiotomy), neonatal resuscitation and perinatal death.

Of the 1,569 participants recruited into the study, 23% had no FGM/C while 77% had FGM/C of varying severity. The risk of postpartum haemorrhage was doubled for women with type I FGM/C, tripled in type II FGM/C and increased by 5-fold for those with type III and IV FGM/C. Caesarean section and perineal tears were also increased. FGM/C was associated with increased risk for neonatal resuscitation and perinatal death.

FGM/C is associated with poor obstetric and neonatal outcomes in the Gambia with degree of risk correlating with the severity of FGM/C.

## Background

The World Health Organization (WHO) definition of Female Genital Mutilation/Cutting (FGM/C) comprises all procedures involving partial or total removal of the external female genitalia or other injury to the female genital organs for non-medical reasons^1^. An estimated 200 million women and girls worldwide have been subjected to this practice, the majority living in African countries^2^. FGM/C spans cultural and ethnic groups and occurs among Muslim, Christian and secular communities. The WHO classifies FGM/C by type and severity of mutilation, Type I being the least mutilating and Type III, also known as infibulation, being the most severe.

Reasons given for the practice of FGM/C are based on cultural and religious beliefs and have no basis in science. These include: prevention of promiscuity in the female, enhancement of male sexual performance and pleasure, maintenance of cleanliness of the genital area, aesthetic reasons, enhancement of fertility and improving a woman's marriage prospects^3^. However, the practice of FGM/C is fraught with adverse health consequences and is frequently performed by traditional practitioners who have had no formal medical training, thus increasing the associated risks 4. Immediate complications include bleeding leading to shock, transmission of infection and injuries to adjacent organs like the urethra and the rectum. Long term sequelae of FGM/C include dyspareunia, anorgasmia, Para clitoral cyst and chronic pain^3^. Obstetric risks include prolonged labour, increased caesarean section rates, increased rates of episiotomy, perineal tears and postpartum haemorrhage^3^–^5^. Women with FGM/C also suffer increased rates of perinatal complications including need for neonatal resuscitation, still birth and early neonatal death^5^.

In part because of these negative health consequences of FGM/C, many governments around the world have outlawed the practice. However, FGM/C continues to be practiced at high rates across many African countries.

The WHO classifies FGM/C into 4 types^1^,^5^. Type I is a partial or complete removal of the clitoris and/or the prepuce (clitoridectomy). In type II, the clitoris and labia minora are partially or completely removed with or without excision of the labia majora. Type III FGM/C involves a narrowing of the vaginal orifice by cutting the labia minora and /or the labia majora and creating a covering seal by close apposition of the cut surfaces. Type IV refers to all other harmful procedures to the female genitalia for non-medical purposes like pricking, cauterization and piercing^1^,^5^. The severity of the FGM/C and its complications worsens from type I to type III.

In 2010 a survey in the Gambia found the national prevalence rate of FGM/C among women aged 15–49 to be 76.3%^6^. The Foundation for Research on Women's Health, Productivity and the Environment (BAFROW) reports that seven of The Gambia's nine ethnic groups practice FGM/C 4. Nearly all Mandinkas, Jolas and Hausas (together 52% of the population) practice Type II on girls between 10 years and 15 years of age. The Sarahulis (9% of the population) practice Type I on girls one week after birth. The Bambaras (1% of the population) practice Type III, which takes place when girls are between 10 years and 15 years of age. The Fulas (18% of the population) engage in a practice analogous to Type III that is described as “vaginal sealing” on girls anywhere between one week and 18 years of age.

Evidence on healthcare outcomes of FGM/C in the Gambia is currently limited. One study found an association between FGM/C and higher rates of bacterial vaginosis and herpes simplex virus 2^7^, but a second study did not uphold this finding^8^. A further study of Gambian women with FGM/C presenting with gynaecological problems attributed many of these to FGM/C^9^. This study did not have a control group and relied on retrospective data in a selected group of women. The same group performed another study of obstetric outcomes of FGM/C, which included women without FGM/C as a control group^10^. This study relied on retrospective reporting of complications by the women but showed significantly worse outcomes for women with FGM/C including prolonged labour, episiotomy, perineal tears, caesarean section, need for neonatal resuscitation and stillbirth. There have been no prospective studies of obstetric outcome of FGM/C in the Gambia.

With a population of just 1.8 million people The Gambia is a small country that falls low on the human development index (165 out of 187 in 2012 11), with high rates of absolute poverty and poor access to healthcare 12. Sadly, it faces multiple challenges in the field of health and typifies many of the issues surrounding the current crisis in human resources for health in Africa. As such, the additional health needs and risks posed by FGM/C are happening against a background of malnutrition, poor maternal and child health indicators and low rates of gender equality 11. In 2015, Gambia's parliament passed a bill outlawing FGM/C for the first time. But with high rates among the current population, it may be many years before the health impact of this ban is felt. National data on healthcare outcomes may be a key component in the advocacy and health education needed to eliminate this practice.

The aims of this study therefore are to determine the obstetric and neonatal outcomes of parturient women with FGM/C in the Gambia.

## Methodology

This was a prospective stratified observational study. Consenting parturient women in the first stage of labour were included in the study. Recruitment was carried out at 4 health facilities across the country: Brikama District Hospital, Jammeh Foundation for Peace Hospital (now Bundung Maternal and child hospital), Bansang Hospital and Edward Francis Small Teaching Hospital.

Four midwives and a medical doctor were employed from each study site and trained in obtaining consent, identifying types of FGM/C, assessing blood loss, Apgar scoring, perineal tear assessment as well as using the questionnaire to gather other relevant data. The midwives were responsible for data collection with supervision from medical doctors and the investigators.

Women who presented in the first stage of labour (before full cervical dilatation) at the 4 participating health facilities during the study period were approached and informed of the study by the study medical doctor or midwife. Trained midwives or medical doctors then examined consenting women who met the eligibility criteria for the presence or absence of FGM/C. Where present, FGM/C was categorized on examination by the WHO classification of the type of FGM/C^5^. Structured questionnaire-based interviews were conducted to obtain demographic information as well as relevant medical and obstetric history. The women were followed up throughout the course of labour and outcome of labour was recorded in the questionnaire.

The eligibility criteria were pregnant women with a singleton foetus presenting in the first stage of labour giving informed consent to be included in the study. Any woman with multiple pregnancy, presenting in the second stage of labour or who was booked for elective caesarean section for any reason was excluded from the study.

The primary outcome was postpartum haemorrhage of >500ml, measured according to the protocol for measuring blood loss in the WHO multicentre randomized trial of misoprostol in the management of the third stage of labour^13^. Secondary obstetric outcomes were: unplanned caesarean section, perineal tears or need for episiotomy. Secondary neonatal outcomes included perinatal death, need for neonatal resuscitation and rates of low birth weight.

The sample size calculation was done using postpartum haemorrhage (binary variable) as the primary outcome. Kaplan et al. reported a proportion of 66.2% of FGM/C I, 26.3% of FGM/C II and 7.5% of FGM/C III^9^. The proportion of women with no FGM/C with postpartum haemorrhage was assumed to be 6% 5. The risk ratio for FGM/C I vs. no FGM/C and FGM/C II vs. no FGM/C was set at 2 while that of FGM/C III vs. no FGM/C was set at 2.5. The type I error was set at 5% and the power at 80%. A sample size ratio of 1 for comparing FGM/C I to no FGM/C and FGM/C II to no FGM/C but a ratio of 1/3 for comparing FGM/C III or IV to no FGM/C because of the low prevalence of FG/C III or IV was considered. A minimum of 1178 parturient women (a minimum of 356 in no FGM/C, FGM/C I and FGM/C II and of 110 in FGM/C III or IV) was the calculated sample size.

Poisson regression was performed to assess the association between FGM/C and binary outcomes. Linear regression model was performed to assess the association between FGM/C and continuous outcomes. Confounders adjusted for were selected based on known associations with the outcome under study or factors that were hypothesized to play a role in the outcome under study in our setting. Data was analysed for test of association using STATA.

The care provided to women and babies was in accordance with normal standards and protocols at the participating health facilities. The Gambian Government Ethics Committee gave approval for the study.

## Results

The total number of deliveries during the 5-month study period (1st May 2016 to 30th September 2016) from all of the participating health facilities was 3,867 out of which 2,197 (56.8%) met the inclusion criteria. However only 1,821 (82.9%) were informed of the study out of which 1,569 (83.7%) consented and were recruited into the study. Of the 1,569 participants that were recruited into the study, 23% had nFGM/C while 77% had FGM/C. Table 1 shows the baseline characteristics of study participants stratified by the presence and type of FGM/C.

**Table 1:** Baseline characteristics of study participants stratified by type of FGM

Variable	No FGM (n=361)	WHO type 1 (n=372)	WHO type 2 (n=704)	WHO type 3 or 4 (n=132) c	p-value
**Age in years**, n (%)					
*Median (1^st^-3^rd^ quartiles)*	27 (22–31)	25 (21–30)	25 (22–30)	24 (20–29)	<0.001#
<20	32 (8.9)	61 (16.4)	89 (12.6)	25 (18.9)	
20–24	94 (26.0)	107 (28.8)	225 (32.0)	50 (37.9)	
25–29	104 (28.8)	84 (22.6)	185 (26.3)	27 (20.5)	
30–34	81 (22.4)	75 (20.2)	118 (16.8)	13 (9.9)	
35 or greater	50 (13.9)	45 (12.1)	87 (12.4)	17 (12.9)	
**Tribe**, n (%)					
Mandinka	32 (8.9)	150 (40.3)	329 (46.7)	60 (45.5)	<0.001*
Fula	45 (12.5)	144 (38.7)	202 (28.7)	32 (24.2)	
Wollof	180 (49.9)	15 (4.0)	23 (3.3)	4 (3.0)	
Jola	25 (6.9)	29 (7.8)	75 (10.7)	13 (9.9)	
Other a	79 (21.9)	84 (9.1)	75 (10.7)	23 (17.4)	
**Education level**, n (%)					
None	148 (41.0)	171 (46.0)	284 (4.03)	50 (37.9)	0.08*
Primary/non-formal	93 (25.8)	102 (27.4)	210 (29.8)	36 (27.3)	
Secondary	94 (26.0)	82 (22.0)	147 (20.9)	38 (28.8)	
Tertiary	26 (7.2)	17 (4.6)	63 (9.0)	8 (6.1)	
**Residential area**, n (%)					
Urban	248 (68.7)	201 (54.0)	431 (61.2)	87 (65.9)	<0.001*
Rural	113 (31.3)	171 (46.0)	273 (38.8)	45 (34.1)	
**Height in cm**, median (1^st^-3^rd^ quartiles) b	162 (158–167)	160 (155–165)	160 (156–165)	160 (156–165)	0.003#
**Parity**					
*Median (1^st^–3^rd^ quartiles)*	2 (0–3)	2 (0–3)	1 (0–3)	0 (0–2)	<0.001#
0	102 (28.3)	121 (32.5)	212 (30.1)	67 (50.8)	
1	67 (18.6)	64 (17.2)	157 (22.3)	28 (21.2)	
2	62 (17.2)	61 (16.4)	121 (17.2)	11 (8.3)	
3	49 (13.6)	45 (12.1)	79 (11.2)	9 (6.8)	
4	40 (11.1)	35 (9.4)	49 (7.0)	5 (3.8)	
5 or greater	41 (11.4)	46 (12.4)	86 (12.2)	12 (9.1)	
**Chronic medical conditions**, n (%)					
Yes	18 (5.0)	29 (7.8)	34 (4.8)	6 (4.6)	0.19*
No	343 (95.0)	343 (92.2)	670 (95.2)	126 (95.4)	
**Previous caesarean section**, n (%)					
Yes	5 (1.4)	21 (5.7)	26 (3.7)	4 (3.0)	0.02
No	356 (98.6)	351 (94.3)	678 (96.3)	128 (97.0)	
**Number of antenatal care visits**, n (%)					
*Median (1^st^–3^rd^ quartiles)*	3 (3–4)	3 (3–4)	3 (3–4)	3 (3–4)	0.42#
0	0 (0.0)	0 (0.0)	2 (0.3)	1 (0.8)	
1	14 (3.9)	18 (4.8)	32 (4.6)	10 (7.6)	
2	50 (13.9)	56 (15.1)	104 (14.8)	15 (11.4)	
3	100 (27.7)	108 (29.0)	187 (26.6)	46 (34.9)	
4 or greater	153 (42.4)	156 (41.9)	308 (43.8)	49 (37.1)	

a include Jahanka, Konyagi, Manjago, Serere, Serahule, Woyinko and other minority tribes.

b Data were missing for 130 women who were excluded from the descriptive analysis.

c Only six FGM type IV were found and recruited into the study.

# Kruskal-Willi's test.

* Pearson chi-square test.

Table 2 shows the obstetric and neonatal outcomes of FGM/C and table 3 shows the association between duration of labour and the type of FGM/C.

**Table 2:** Crude and adjusted relative risk (RR) of adverse outcomes for any type of FGM vs No FGM

Outcome and FGM status	Cases/population	Prevalence (%)	Crude RR (95% CI) *	p	Adjusted RR (95% CI) *	p
**Obstetric outcomes**						
Post-partum blood						
loss >500ml						
No FGM	34/361	9.4	1.0	<0.001	1.0	<0.001†
FGM I	67/372	18.0	1.9 (1.3–2.8)	2.1 (1.3–3.5)		
FGM II	161/704	22.9	2.4 (1.7–3.4)	2.3 (1.7–3.9)		
FGM III	58/126	46.0	3.1 (2.3–4.2)	2.9 (2.1–5.8)		
Caesarean section						
No FGM	16/361	4.4	1.0	0.004	1.0	0.036††
FGM I	36/372	9.7	2.2 (1.2–3.9)	2.1 (1.2–5.1)		
FGM II	81/704	11.5	2.6 (1.5–4.4)	2.4 (1.1–4.8)		
FGM III	16/126	12.7	2.9 (1.5–5.6)	2.7 (1.2–6.1)		
Episiotomy or perineal tear						
No FGM	76/361	21.1	1.0	<0.001	1.0	<0.001†††
FGM I	140/372	37.6	1.8 (1.4–2.3)	1.7 (1.3–2.1)		
FGM II	283/704	40.2	1.9 (1.5–2.4)	1.8 (1.4–2.2)		
FGM III	101/126	80.2	3.8 (3.1–4.7)	2.4 (2.1–3.4)		
**Fetal outcomes**						
Perinatal death						
No FGM	7/361	1.9	1.0	0.13	1.0	0.11*
FGM I	13/372	3.5	1.8 (0.7–4.5)	1.9 (0.7–4.6)		
FGM II	33/704	4.7	2.4 (1.1–5.4)	2.5 (1.1–5.7)		
FGM III	3/126	2.4	1.2 (0.3–4.7)	1.3 (0.3–5.1)		
Need for resuscitation						
No FGM	31/361	8.6	1.0	<0.001	1.0	<0.001**
FGM I	50/372	13.4	1.6 (1.0–2.4)	1.9 (1.2–3.2)		
FGM II	121/704	17.2	2.0 (1.4–2.9)	2.5 (1.6–4.0)		
FGM III	37/126	29.4	3.4 (2.2–5.3)	3.9 (2.4–6.5)		
Birth weight <2500g						
No FGM	30/361	8.3	1.0	0.99	1.0	0.98***
FGM I	29/372	7.8	0.9 (0.6–1.5)	0.9 (0.6–1.5)		
FGM II	57/704	8.1	1.0 (0.6–1.5)	0.9 (0.6–1.5)		
FGM III	11/126	8.7	1.1 (0.5–2.0)	0.9 (0.5–1.7)		

* 95% confidence interval of the relative risk.

† Adjusted for parity and birth weight

†† Adjusted for parity, education, birth weight and previous caesarean section

††† Adjusted for parity, maternal age, birth weight and instrumental delivery

* Adjusted for maternal age, residence and history of chronic illness.

** Adjusted for maternal age, residence, parity and history of chronic illness

*** Adjusted for parity, residence and history of chronic illness

**Table 3:** Duration of labour (in hours) and FGM/C type

FGM status	Geometric mean	Crude geometric mean ratio (95% CI) (95% CI) *	Adjusted geometric mean ratio (95% CI) **
No FGM	5.9	1.0	1.0
FGM I	8.3	1.4 (1.3–1.5)	1.4 (1.3–1.6)
FGM II	8.9	1.5 (1.4–1.6)	1.6 (1.4–1.7)
FGM III	10.6	1.8 (1.6–2.0)	1.8 (1.6–2.1)

* p-value = <0.001

** p-value = <0.001; Adjusted for parity, birth weight and maternal body mass index

These data show that all forms of FGM/C increase the risk of poor obstetric outcomes: postpartum haemorrhage (blood loss of over 500ml), caesarean section and episiotomy or perineal tear. Neonatal outcomes are similarly shown to be poor for women with FGM/C; there was increased risk of need for neonatal resuscitation with all forms of FGM/C and perinatal death increased in type II FGM/C. In types I and III FGM/C an increased number of perinatal deaths were observed compared with no FGM/C but these numbers were too small to demonstrate statistical significance. There was no statistically significant difference in risk of having a baby with birth weight under 2500g in women with FGM/C.

## Discussion

Across all outcomes, as the type of FGM/C moves from type I to type III (corresponding with an increase in the severity of mutilation) then the risks increase further, adding weight to the argument that the association is causal. These findings are in keeping with international data on outcomes of FGM/C in African countries^5^.

Although no statistical significance in perinatal death among women with FGM/C type I and III was observed, this has been shown in a large study with similar settings^5^. Despite being a relatively small study, it was possible to show increased perinatal death in FGM/C type II, with a two-fold increase in risk. It may be that the study was underpowered to demonstrate increased perinatal death in the other types of FGM/C. The increased rate of need for resuscitation is similarly striking and is likely to correlate to increased rates of hypoxic ischaemic encephalopathy (HIE) in babies who do survive with subsequent long-term disability.

This study was carried out at four health-care facilities across the country of the Gambia. As such there was a good representation of the tribal spread and the aim was to proportionally represent the varied population of the country.

There are multiple challenges around conducting research into FGM/C in this setting. Among communities where this is practiced there is an understandable reluctance to discuss the issue. There may be stigma in acknowledging that FGM/C has been performed, or that health consequences occur. As such it is difficult to exclude the risk of recruitment bias for those women willing to join the study. However, the use of local midwives consenting women and performing data collection at each site aimed to ameliorate this risk.

Many of the population lack the means to travel or seek emergency help when required. As such richer women and those with access to healthcare may be over-represented in this study. Similarly, women with pregnancy complications or risk of complex delivery may preferentially seek out healthcare settings and be overrepresented in this study. This is important in interpreting our findings because while 86.2% of pregnant women in the Gambia receive antenatal care from a skilled health professional, only 57.2% of all deliveries are conducted by a skilled health professional^14^. A weakness of this study was that not all eligible women presenting to the healthcare facilities during the study window were approached potentially introducing selection bias. Limited resources and availability of staff made this challenging.

A limitation of all studies of medical outcomes of FGM/C is that they are observational (i.e., it is not possible to randomize). As such it is not possible to draw definitive conclusions about causality and to exclude confounding factors. However, it was only after recruitment into the study that the women were examined for the presence of FGM/C thus ensuring that the groups were as similar as possible. Another limitation of our study is the absence of data on de-infibulation before delivery in type III FGM/C. WHO guidelines suggests that antepartum or intrapartum de-infibulation be done for women with type III FGM/C^15^. However, de-infibulation is not mentioned in antenatal care guidelines in the Gambia and there is no data on its routine practice.

The outcomes of need for episiotomy and caesarean section may represent a source of bias. The medical decision to progress to such measures had a subjective element and may show baseline variation in practice between settings. Healthcare worker's experience of FGM/C obstructing labour may lead to increased willingness to progress to these measures when FGM/C is noted to be present.

Studies on obstetric outcomes of FGM/C show that outcomes are worse in resource poor settings whereas there was no significant difference in obstetric outcomes in resource rich countries^16^–^19^. This is probably due to a lack of knowledge and access to antenatal care services in resource poor countries^16^. Even though The Gambia is a resource constrained country, more than 85% of pregnant women receive antenatal care from a skilled car provider^14^ and the country has more than the required WHO minimum number of recommended comprehensive emergency obstetric care centres^20^,^21^. However, the quality of care available in these health centres needs to be evaluated.

In conclusion, FGM/C in The Gambia was found to be associated with adverse obstetric outcomes, risk of postpartum haemorrhage, unplanned caesarean section, risk of perineal tears and episiotomy. In terms of neonatal outcomes FGM/C is associated with increased risk of need for neonatal resuscitation and FGM/C type II is associated with increased perinatal death. Across all outcomes, as the type of FGM/C moves from type I to type III (corresponding with an increase in the severity of mutilation) then the risks increase further, adding weight to the argument that the association is causal. FGM/C in The Gambia was not shown to be associated with low-birth-weight babies in this study. We recommend that routine vaginal examination be included as part of the first antenatal care visit in The Gambia and women who are found to have type III FGM/C be referred to health facilities were de-infibulation can be done before delivery. FGM/C has been banned in The Gambia but there is fear that it may be driven underground and continue at high rates. It is hoped that the results of this study will be useful in the advocacy and sensitization of Gambians needed to end this practice. More qualitative research will be needed to understand the factors that are driving the practice in order to finally eliminate it from the Gambia.

## Declarations

### Availability of data and material

The datasets generated and/or analysed during this study are available from Action Aid International The Gambia on reasonable request.

## Authors' Information

PI is an Obstetrician/Gynaecologist at EFSTH.

## List Of Abbreviations

EFSTH: Edward Francis Small teaching Hospital.
FGM: Female genital mutilation
FGM/C: Female genital mutilation or cutting
HIV: Human immunodeficiency virus
LBW: Low birth weight
WHO: World Health Organization
